# Can the Germ of Measles Remain Virulent for a Long Time?

**Published:** 1914-08

**Authors:** 


### ABSTRACTS

**Can the Germ of Measles Remain Virulent for a Long Time?** Sakkorafos, recites in Le Progres Médical, 1914, No. 21, the case of a lady who contracted measles ca. four months after the death of her child from this disease, after opening the box in which she had put away the playthings of this child. Any other source of infection can be excluded.—C. G. L-W.

